# Insect Responses to Linearly Polarized Reflections: Orphan Behaviors Without Neural Circuits

**DOI:** 10.3389/fncel.2018.00050

**Published:** 2018-03-20

**Authors:** Tanja Heinloth, Juliane Uhlhorn, Mathias F. Wernet

**Affiliations:** Division of Neurobiology, Institut für Biology, Fachbereich Biologie, Chemie & Pharmazie, Freie Universität Berlin, Berlin, Germany

**Keywords:** insect vision, polarized light, behavior, orientation, water detection, neuroethology, visual ecology, neural circuits

## Abstract

The e-vector orientation of linearly polarized light represents an important visual stimulus for many insects. Especially the detection of polarized skylight by many navigating insect species is known to improve their orientation skills. While great progress has been made towards describing both the anatomy and function of neural circuit elements mediating behaviors related to navigation, relatively little is known about how insects perceive non-celestial polarized light stimuli, like reflections off water, leaves, or shiny body surfaces. Work on different species suggests that these behaviors are not mediated by the “Dorsal Rim Area” (DRA), a specialized region in the dorsal periphery of the adult compound eye, where ommatidia contain highly polarization-sensitive photoreceptor cells whose receptive fields point towards the sky. So far, only few cases of polarization-sensitive photoreceptors have been described in the ventral periphery of the insect retina. Furthermore, both the structure and function of those neural circuits connecting to these photoreceptor inputs remain largely uncharacterized. Here we review the known data on non-celestial polarization vision from different insect species (dragonflies, butterflies, beetles, bugs and flies) and present three well-characterized examples for functionally specialized non-DRA detectors from different insects that seem perfectly suited for mediating such behaviors. Finally, using recent advances from circuit dissection in *Drosophila melanogaster*, we discuss what types of potential candidate neurons could be involved in forming the underlying neural circuitry mediating non-celestial polarization vision.

## Introduction

Across insect species, a great diversity of photosensitive, image-forming structures (eyes) has been described which allow for visually guided navigation during daytime under bright illumination, as well as around dusk or dawn, or even at very low light intensities during the moonlit night (Land and Fernald, 1992). The mechanisms underlying both the sensation and subsequent integration of different aspects of the visual world, like intensity, contrast, motion, or color are crucial for shaping the specific behavioral repertoires of different animal species. One well-studied example is the perception of linearly polarized light, a sensory ability that is common to some vertebrates (birds, fishes), as well as marine invertebrates (Cephalopods, Crustaceans), and many insects (Nilsson and Warrant, 1999; Cronin et al., 2003; Mathejczyk and Wernet, 2017). Initially, sunlight (or moonlight) is unpolarized and manifests a randomly distributed e-vector, but atmospheric scattering produces a celestial e-vector pattern that changes during the course of the day. Hence, polarized skylight represents wide-field celestial cue for navigation (for instance when the celestial body is obstructed from view), used by many insects: “Truly navigating” central place forager species like honeybees or desert ants certainly manifest the most impressive navigational skills (from their hive/nest to a food source and back), whereas other insect species appear to use celestial polarization for more basic orientation tasks (for instance crickets or dung beetles; Labhart and Wehner, 2006; Homberg, 2015). Reflection of sunlight off shiny surfaces (water, leaves, or body surfaces) represents the second important source of polarized light found in nature (Wehner, 2001). Such polarized reflections (always horizontally polarized, in the case of water bodies) can be used to either seek out or avoid localized water sources (ponds, lakes), or to follow the course of a continuous stream (creeks, rivers). Studies on many different insect species have shown that polarized reflections also provide important information for evaluating the quality of certain environments, for instance as suitable food source, or oviposition sites (Wehner, 2001). Similarly, polarized reflections off shiny body surfaces can be used to identify both conspecifics (for instance during courtship), as well as prey (in the case of certain blood-sucking insects). Conversely, the glare resulting from polarized reflections can be a nuisance to insects living on the water surface, resulting in mechanisms to specifically filter it out. In the “Behavioral Responses of Different Insect Species to Reflected Linearly Polarized Light” section we present an overview over the growing number of insect species that manifest specific behavioral responses to linearly polarized reflections.

The necessary substrate for polarization sensitivity in the insect retina is formed by a specialized ultrastructure of the photoreceptor light-gathering membranes, the so-called rhabdomeres (Wehner, 1976). Usually eight or nine photoreceptor neurons (in some species even more) are organized within stereotypical unit eyes, or ommatidia, varying numbers of which together form the insect retina (Wernet et al., 2015). Specialized ommatidia in the “Dorsal Rim Area” (DRA) of many insect eyes contain highly polarization-sensitive photoreceptors that have been identified as the substrate for detecting linearly polarized skylight (for review see Labhart and Meyer, 1999). In the DRA, two groups of photoreceptor cells from the same ommatidium have rhabdomeres with straightly aligned microvillar membranes. Such a design is crucial for achieving high polarization sensitivity, since the rhodopsin molecules appear to be anchored in a fixed orientation along the axis of these membranes, leading to preferential absorption of light of a specific e-vector orientation (Roberts et al., 2011). Outside the DRA region, polarization sensitivity is often suppressed through rhabdomere twisting (i.e., the rhabdomere orientation changes as a function of the depth through the retina), thereby avoiding mixture of color and polarization information within the same photoreceptor cell (Wehner and Bernard, 1993). In the DRA, the two groups of untwisted, polarization-sensitive photoreceptors manifest preferred e-vector orientations that are orthogonal to each other (Labhart and Meyer, 1999), a design that is optimal for polarization-opponent coding (Labhart, 1988; Heras and Laughlin, 2017). Furthermore, they always express the same Rhodopsin molecules thereby again avoiding confusion between color and polarized light information. Interestingly, the wavelength sensitivity of polarization-sensitive DRA photoreceptors varies between species, most likely reflecting their different life styles (Barta and Horváth, 2004; Hegedüs et al., 2006a): UV-sensitive receptors are found in bees, ants, flies, butterflies and some beetles (Vonhelversen and Edrich, 1974; Labhart, 1986; Fortini and Rubin, 1990); blue-sensitive rhodopsins in the DRA of crickets and locusts (Henze et al., 2012; Schmeling et al., 2014); green-sensitive DRA photoreceptors are used by cockchafers and the European corn borer moth *Ostrinia nubialis* (Labhart et al., 1992; Belušič et al., 2017). At the ventral rim of the insect retina there exists no specialized type of polarization-sensitive ommatidia analogous to the DRA, which would be common to all insects. Despite the growing list of reports describing behavioral responses to reflected polarized light, the retinal substrate mediating these responses remains elusive, for the large part. In fact, only three well-documented examples exist demonstrating the existence of photoreceptor cells with specialized rhabdomere ultrastructure at the ventral periphery of insect eyes (from water striders, back swimmers and long-legged flies). Interestingly, the organization and extent of the retinal area harboring specialized photoreceptors for ventral polarization vision differs greatly between these three examples: either (i) the entire ventral retina manifests specialized photoreceptor ultrastracture (with a sharp boundary to the rest of the eye); or (ii) discrete zones within the ventral half of the retina show different specializations; or (iii) alternating stripes of ommatidia, each containing pairs of photoreceptors manifesting two characteristic rhabdomere orientations vis-à-vis each other (either orthogonal or parallel) that are characteristic for each row. In the “Ommatidial Subtypes in the Ventral Insect Retina with Increased Polarization Sensitivity” section, we will compare these three examples and discuss the different use by the animals, as well as their relevance for a life in their respective habitats.

The signals from polarization-sensitive photoreceptors are collected and further processed by the underlying circuits within the optic lobes and the central brain. Both anatomical and electrophysiological studies in several insect species (most prominently: the desert locust) have revealed numerous cell types that show very specific responses to linearly polarized light. In the case of polarized skylight detected by the DRA, a neuronal “compass” pathway was reconstructed, leading from the DRA ommatidia to the central complex, via an optic glomerulus called the anterior optic tubercle (Homberg et al., 2011; Homberg, 2015). Over the past decades, work in this field has provided exciting insight into how e-vectors are detected and processed into polarization-opponent signals that become modulated with respect to the time of day (in a process referred to time compensation), ultimately lading to a map-like representation of different e-vectors within columnar structures of the central complex (Sakura et al., 2006; Heinze and Homberg, 2007; Kinoshita et al., 2007; Heinze and Reppert, 2011; Homberg et al., 2011; el Jundi et al., 2015). Considering this high degree of detail, it is quite shocking that virtually nothing is known about the neural circuits processing polarized reflections detected by specialized ommatidia in the ventral periphery of the insect retina. Systematic approaches towards characterizing most, if not all neuronal subtypes in the fruit fly brain provide one attractive way towards characterizing these elusive circuit elements (Pfeiffer et al., 2008). Interestingly, two independent studies have demonstrated that fruit flies can detect linearly polarized light when presented to the ventral half of the retina, by analyzing spontaneous alignment of the body axis with respect to the incident e-vector (polarotaxis; Wolf et al., 1980; Wernet et al., 2012). Surprisingly, these responses appear to be mediated by only one of the two ommatidial subtypes that are randomly distributed throughout the fly retina. However, an incomplete ultrastructure analysis of only a small sample from this ommatidial subtype called “pale” revealed no subtype-specific rhabdomere untwisting indicative of high polarization sensitivity (Wernet et al., 2012). Nevertheless, a different study revealed a specific role for the other stochastic ommatidial subtype (called “yellow”) in mediating color discrimination (Schnaitmann et al., 2013). It remains an open question whether “pale” and “yellow” ommatidia (found across fly species) could indeed serve different functions like polarization vs. color vision. Nevertheless, the fly retinal mosaic of randomly distributed ommatidial subtypes provides an attractive model for investigating differences in the cellular composition of their downstream circuits. In the “Neural Circuits Connecting to Specific Ommatidial Subtypes—Lessons from *Drosophila*” section, we will summarize the growing data on the neuronal subtypes that are specific to “pale” or “yellow” ommatidia in *Drosophila*, as well as the developmental mechanisms leading to subtype-specific connectivity. Even if serving a different function, the logic behind forming “pale” vs. “yellow” specific differences in circuitry could serve as a model for how distinct polarization vision circuit elements are specified at the ventral periphery of the insect eye.

## Behavioral Responses of Different Insect Species to Reflected Linearly Polarized Light

**Figure 1:**
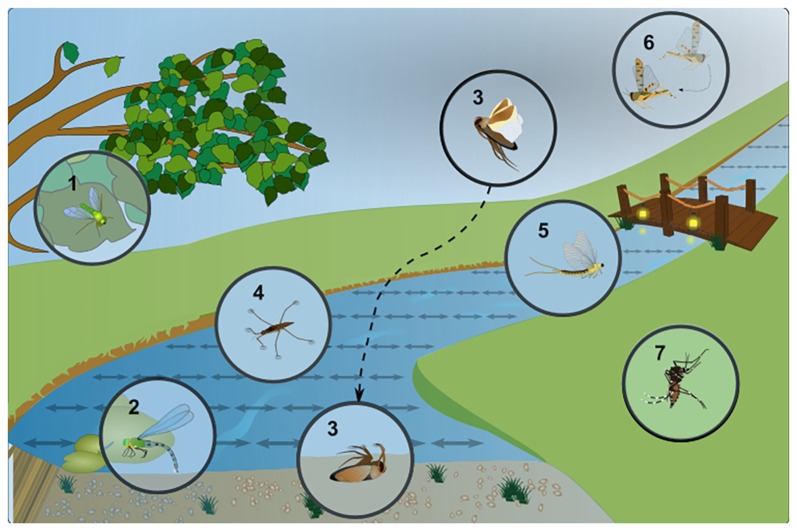
Examples of insect responses to linearly polarized light reflected off water. Specific adaptions of different semi-aquatic insect species to a life in close proximity to water bodies and their characteristic behavior in response to linearly polarized, shiny surfaces (symbolized by double-headed arrows). (1) Different species of long-legged dipteran flies (Dolichopodidae) can be found close to the water, hunting for prey on the water surface, which produces strong glare due to polarized reflections. (2) Dragonflies are known to oviposit (lay their eggs) onto the water surface, or in some cases on any shiny surface they mistake for water. (3) The “back swimmer” *Notonecta glauca*, a hemipteran bug shows a characteristic “plunge reaction” into water (or linearly polarized surfaces). It then spends a considerable part of its life hanging under the water surface hunting for prey. (4) Another hemipteran, the water strider *Gerris lacustris* is constantly faced with the surface glare of polarized reflections, making it more difficult to identify features under water. (5) During their “dispersal flight” after copulation, female Mayflies (Ephemeroptera) are known to follow a river upstream, to find an oviposition site. Linearly polarized reflections have been identified as a major guiding cue during this process and unpolarized light pollution (for instance at illuminated bridges) forms a major obstacle. (6) Flying desert locusts (*Schistocerca gregaria*) are repelled by linearly polarized reflections, most likely to avoid crash-landing in the sea. (7) Different mosquito species, as well as certain midges (all Nematocera), seem attracted to water surfaces via their linearly polarized reflections. However, this effect seems to be rather minor in some cases, since olfactory stimuli dominate.

When reflected off a shiny, flat and non-metallic surface like water, sunlight becomes horizontally polarized, with the maximum degree of polarization occurring at an angle of incidence of 53^°^ (for an air/water interface), also known as “Brewster’s angle”. Different flying insects appear to use polarized reflections to identify water bodies (Wehner, 2001; summarized in Figure 1). Depending on the species studied, such polarized reflections can be attractive, as well as repulsive, since swarms of flying desert locusts were shown to avoid flying over polarized surfaces, probably to avoid crash-landing over sea (Shashar et al., 2005). Probably the best studied example of any water-seeking insect attracted to polarized surfaces is the hemipteran back swimmer *Notonecta glauca*. This bug visually identifies water surfaces when conducting dispersal flights between water bodies, resulting in a characteristic diving reaction during which the animal raises its body axis to an angle of 53 degrees shortly before diving into the water (Schwind, 1984; Wehner, 1987). Horizontal platforms emitting linearly polarized UV light are sufficient to induce this diving reaction (Schwind, 1983a). Interestingly, Notonecta spends much of its lifetime hanging under the water surface, from where it observes the airy world above. Hence its visual system needs to accommodate both sensitivity to horizontally polarized light, as well as accurate vision through the water/air interface, which is reflected by the separation of its ventral retina into discrete zones (as discussed in the “Ommatidial Subtypes in the Ventral Insect Retina with Increased Polarization Sensitivity” section). It is known that females from many different semi-aquatic insect species erroneously lay their eggs on shiny surfaces that they seem to have mistook for water. Examples are parked cars, black gravestones, glass buildings, and sometimes even oil pits (Horváth et al., 1998, 2007; Kriska et al., 1998, 2008, 2007). One fly species, *Halaeomyia petrolei* even became adapted to a life near (or in the case of its larvae/pupae: inside) naturally occurring petroleum pools, feeding on arthropod prey that became trapped there (Thorpe, 1930). Female mayflies were shown to use horizontally polarized reflections off water to direct their so-called “compensatory upstream flights” before oviposition (Farkas et al., 2016) and this dispersion behavior is disrupted by (unpolarized) light pollution, for instance illuminated bridges (Szaz et al., 2015). In another example, female dragonflies attempted to lay eggs on an artificial, horizontally polarized surface, assuming it to be water (Wildermuth, 1998). Similarly, male dragonflies approach polarized surfaces to establish an aquatic territory, hence in this case both sexes show strong responses to reflected polarized light. At this point it remains unclear whether insects can distinguish different degrees of polarization (a stimulus that is 100% polarized virtually never occurs in nature). For instance, it was proposed that dragonflies could use such information to distinguish between habitats, for instance dark and light ponds since the degree of polarization (i.e., the ratio between reflected, polarized light and scattered, unpolarized light) is proportional to the absorbance of water in the pond and to the amount of organic nutrients suspended in water (Bernáth et al., 2002). Interestingly, visual cues like polarized reflections seem to play a rather minor role for female mosquitoes when identifying oviposition sites after a blood meal. Instead, chemical cues indicating the presence of conspecifics, eggs, or larvae appear to strongly dominate (Bernáth et al., 2008, 2012). However, one recent study confirmed polarization sensitivity of the ventral half of the retina for the Zika virus transmitting species *Aedes aegypti*. In these experiments, an optomotor reaction to rotating stripes of alternating orthogonal directions of polarization was demonstrated (Bernáth and Meyer-Rochow, 2016). Hence, it appears that mosquito polarization vision may usually be masked by the chemical senses and it remains possible that plays a role only under very specific behavioral conditions (Bernáth et al., 2012). Interestingly, the related non-biting midges (Chironomidae) which have a comparable lifestyle appear to rely more heavily on visual cues for the detection of water surfaces (Lerner et al., 2008; Horváth et al., 2011). It must be noted that polarized reflections off water can also be problematic for many (semi-)aquatic insects: for instance, the resulting glare interferes with observing underwater objects from above the water surface (Wehner, 2001). This can be particularly relevant for species living directly on the water surface, like water striders (*Gerris lacustris*), or certain flies hunting for prey living on the water surface (like Doliochopodidae). Specific retinal adaptations found in these species could therefore aim at filtering out this stimulus (as we will discuss in the “Ommatidial Subtypes in the Ventral Insect Retina with Increased Polarization Sensitivity” section).

**Figure 2:**
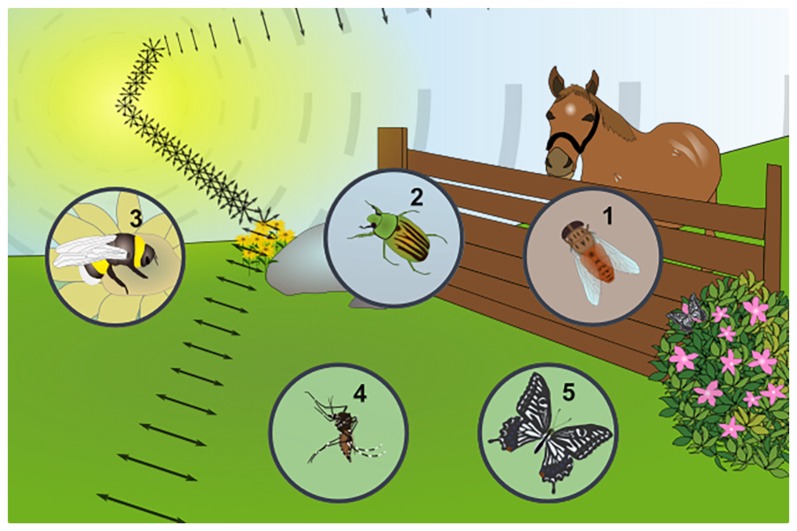
Examples of insect responses to linearly polarized reflections from other substrates. Any shiny, non-metallic surface can produce linearly polarized reflections from unpolarized sunlight, as shown for the example of a flower, where both flowers and leaves can produce this stimulus (symbolized by the double headed arrows) and carry different kinds of information for insects. (1) Blood-sucking horse flies (*Tabanidae*) are strongly attracted by objects reflecting linearly polarized light (a fact exploited in horse-fly traps). The facts that horses and cattle reflect linearly polarized light is in agreement with these insects using this stimulus to detect their prey. (2) The exocuticle of several species of scarab beetles (*Coleoptera*) was shown to reflect circularly polarized light, yet it remains unclear whether this stimulus can be perceived by the animals (i.e., rightward- vs. leftward circularly polarized light), since contradicting behavioral studies exist. (3) Bumblebees (*Hymenoptera*) can be trained to learn different patterns of polarized light, reminiscent of the patterns that could be produced by blooming flowers, suggesting this stimulus may influence their pollenating activity. (4) It is unlikely that female mosquitoes (Nematocera) are attracted by linearly polarized reflections off the body surface of their prey and olfactory stimuli (sweat, CO_2_) clearly dominate. Nevertheless, optomotor responses to alternating stripes of orthogonally oriented polarization demonstrate the existence of polarization sensitivity in the ventral half of the retina. (5) Linearly polarized reflections represent an important stimulus for different butterfly species (*Lepidoptera*): for instance, reflections off the body surface of conspecifics are an important cue for identifying potential mates in an otherwise cluttered, optically rich environment. Furthermore, reflections off leaves bear important information about how well-suited they are as an oviposition site.

Of course linearly polarized reflections can be produced by any shiny, non-metallic object and many insects have been shown to detect such stimuli (summarized in Figure 2). For instance, shiny leaves are an attractive oviposition cue for certain butterfly species (Kelber, 1999a,b). Interestingly, female butterflies most likely perceive “false colors”, since their visual system is mixing e-vector orientation and information about wavelength. This way, female butterflies can distinguish matte from shiny leaves by perceiving them as different colors (Kelber et al., 2001). Such a system is suitable to evaluate different features, like quality of the landing site (leaf orientation), food quality (for caterpillar offspring), or protection for the eggs. Similar mixing of linear polarization and the intensity of light was also shown in butterflies (Kinoshita et al., 2011), in this case resulting in the perception of differently polarized surfaces as differing in brightness. The wings of many butterflies also produce linearly polarized reflections that can serve as mating signals for conspecifics (Sweeney et al., 2003; Yoshioka and Kinoshita, 2007; Stavenga et al., 2012). Heliconius butterflies most likely use these reflections to increase their visibility in the midst of highly complex visual environment (Douglas et al., 2007). Hence, polarized reflections are used in this case to increase the perceived visual contrast. Some true flies (Diptera) not only show strong attraction to polarized surfaces, but also linearly polarized objects, which was demonstrated for blood-sucking horse flies (Tabanidae; Horváth et al., 2008; Egri et al., 2013). Some of these behaviors are most likely involved in prey detection since polarimetric imaging of horses and cattle reveals strong linearly polarized reflections off their fur (Horváth et al., 2010). Brown and black fur produces the strongest polarized reflections, while the scattering effect of white fur or certain fur patterns like stripes (zebras) and spots (cows) appear to be a suitable protection against horse fly attacks (Blahó et al., 2012a; Egri et al., 2012). An even more sophisticated example for learning to distinguish between different patterns of linearly polarized light comes from bumblebees: it appears that pollinators may also use polarized reflections to identify or evaluate floral targets (Foster et al., 2014). Finally, in a less well understood example, the body cuticle of some scarab beetles were shown to reflect circularly polarized light (Hegedüs et al., 2006b; Jewell et al., 2007; Sharma et al., 2009). This stimulus (an e-vector rotating as the beam of light propagates) would appear unpolarized to most insects, since all e-vector orientations are equally represented as they hit a photoreceptor (Labhart, 1996; Henze and Labhart, 2007). Nevertheless, one study reported specific phototactic responses to circularly polarized light were reported for the scarab beetle *Chrysina gloriosa*, whose body surface produces strong circularly polarized reflections (Brady and Cummings, 2010). Interestingly, the closely related species *Chrysina woodii*, whose cuticle manifests only weak circularly polarized reflections, exhibited no phototactic discrimination between linearly and circularly polarized stimuli. However, it must be noted that another study investigating four different scarab beetle species with well-documented circularly polarized reflections off their exocuticle found no evidence for specific behavioral responses to circularly polarized light (Blahó et al., 2012b). Taken together, a great variety of behavioral responses to reflected polarized light has been described across insect species, affecting very different aspects of their respective ecology and life cycle.

## Ommatidial Subtypes in the Ventral Insect Retina With Increased Polarization Sensitivity

Insect retinas are composed of repetitive unit eyes (ommatidia) which usually contain eight or nine photoreceptor neurons (Wernet et al., 2015). In many cases, specialized ommatidia containing photoreceptors with increased polarization sensitivity can be found in the dorsal periphery, a region called the DRA (Labhart and Meyer, 1999). Only there, pairs of untwisted photoreceptor rhabdomeres within each ommatidium form orthogonal analyzers and gradual differences between neighboring DRA ommatidia are in turn forming a fan-shaped array of analyzers across the DRA. This structure acts as the retinal substrate for detecting the e-vector orientation of the celestial polarization pattern, which the animal can use for improving its navigational skills (Blum and Labhart, 2000; Homberg and Paech, 2002; Wernet et al., 2012; Weir et al., 2016). Although both structure and function of the insect DRA, as well as its downstream circuitry have been described in great detail, much less is known about polarization-sensitive photoreceptors in the ventral periphery of the retina. Most importantly, there exists no specialized type of ommatidia at the ventral rim of the retina with polarization-sensitive photoreceptors for mediating responses to linearly polarized reflections that would be common across insects. Despite the numerous examples for behavioral responses to such stimuli, it is therefore surprising that only three retinal specializations have so far been characterized in the ventral periphery of different insect eyes (see below). For each case, a different design principle is responsible for adapting the ventral retina to the ecological needs of the animal: either specialized ommatidia can be organized as a homogeneous ventral region (*Gerris lacustris*), or subdivided into separate zones (*Notonecta glauca*), or even into alternating rows of ommatidial subtypes (Dolichopodidae).

**Figure 3:**
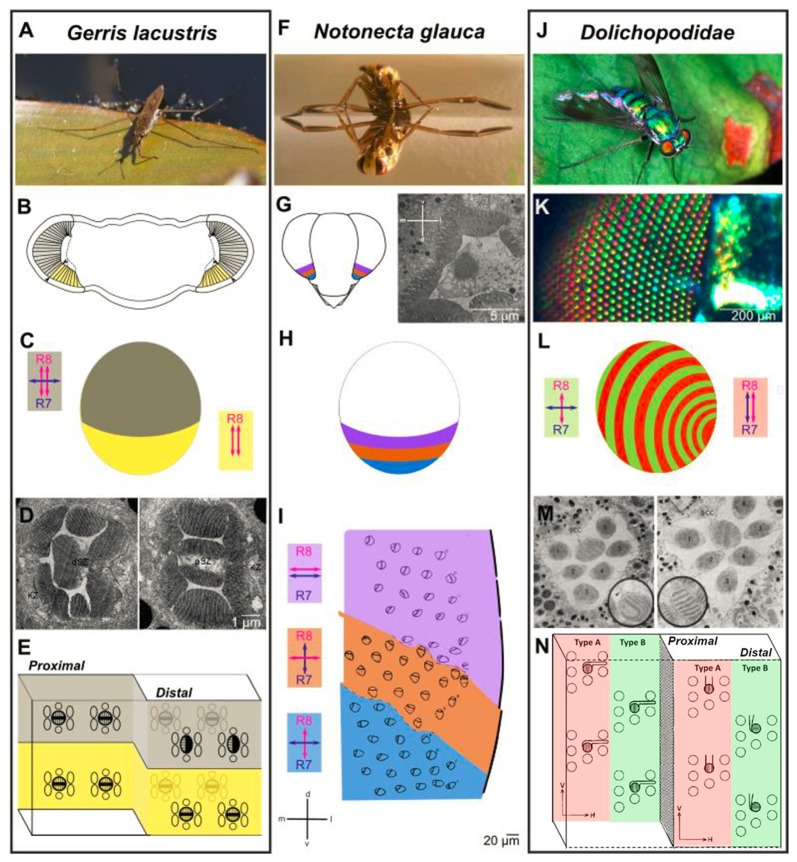
Retinal specialization in the ventral periphery of three semi-aquatic insect species. Investigation of retinal ultrastructure using electron microscopy has revealed three very informative examples for specializations in the ventral periphery of the insect retina, each providing unique adaptations to the life close to linearly polarized water surfaces. **(A–E)** The ventral retina of the water strider *Gerris lacustris* **(A)** is perfectly adapted for filtering out horizontally polarized surface glare. A morphologically distinct ventral region is clearly visible (**B**, marked in yellow). The cellular composition of ommatidia changes drastically at the boundary between dorsomedial and ventral retina: only in the ventral part, the proximal cell with two vertically oriented rhabdomeres spans the entire thickness of the retina (double headed red arrows in **B**), whereas an additional, distal cell with one horizontally oriented rhabdomere is found on top of the distal cell across the dorsomedial retinal field (double headed blue arrow in **B**). Electron microscopy sections through the distal part of the *Gerris* retina shown in **(D)**, with a dorsal ommatidium on the left, and a ventral ommatidium on the right. Summary of cells and rhabdomere orientations at the interface of dorsal and ventral *Gerris* ommatidia shown in **(E)**. **(F–I)** Zonation of the ventral retina in the back swimmer *Notonecta glauca* **(F)** Three distinct zones can be distinguished within the ventral retina of Notonecta, based on the rhabdomere orientations of the inner photoreceptors (named R7 and R8, according to *Drosophila* nomenclature), visualized by electron microscopy **(G)**. Orientation of R7 vs. R8 rhabdomeres differ from parallel and horizontal (most dorsally), to different orthogonal configurations **(H)**. The relative position of the three zones within the ventral retina and their inner photoreceptor rhabdomere orientations are shown in **(I)**. **(J–N)** Alternating rows of ommatidial subtypes in long-legged flies (Dolichopodidae): alternating rows of shiny red and green colored facets in *Dolichopus nitidus* shown in **(K)**. Analysis of retinal ultrastrure using electron microscopy revealed specific differences in R7 vs. R8 rhabdomere orientation between “Type A” ommatidia (red) and “Type B” ommatidia (green): parallel and vertically oriented (Type A) vs. orthogonal (Type B) shown in **(L)**. This subtype-specific difference is achieved by alternating changes in R7 cell rhabdomere orientation. An electron microscopy section through a Type B ommatidium (left) and a Type A ommatidium (right) in **(M)**. **(A,F,J)** Reproduced from Wikimedia under Creative Commons licenses; **(K)** reproduced from (Stavenga et al., 2017) under Creative Commons licenses; **(D)** reproduced with permission from (Schneider and Langer, 1969); **(I)** reproduced with permission from (Schwind, 1983b); **(M)** and **(N)** reproduced with permission from (Trujillo-Cenóz and Bernard, 1972).

In the retina of the hemipteran water strider *Gerris lacustris*, ommatidia in the ventral zone of the adult eye show characteristic morphological specializations: only there, one of the two central cells is lost and the proximal cell extends through the entire retina (Schneider and Langer, 1969; Figures 3A–E). Curiously, this single cell forms two untwisted rhabdomeres, which are both oriented along the dorsoventral axis of the animal. This unidirectional design (as opposed to orthogonal analyzers) is ideal for filtering out polarized reflections, since the resulting glare might represent a challenge for any animal living on the water surface. Hence, such a ventral adaptation forms a “matched filter” which equips the animal with an improved ability to look deeper into the water (Wehner, 1987). Alternatively, it can serve to increase contrast when observing animals against the glare that results from polarized reflections (Schneider and Langer, 1969). Hence, in analogy to the insect DRA, the ventral ommatidia from *Gerris* are morphologically specialized, forming a region with a sharp boundary to the rest of the retina. Only in this ventral region, identified photoreceptor subsets manifest important morphological features with regard to polarization sensitivity.

The second, very well-characterized example for polarization-sensitive photoreceptors at the ventral rim of the insect retina is the hemipteran back swimmer *Notonecta glauca* (Figures 3F–I). In this case, different zones within the ventral periphery of the retina can be distinguished, covering different areas of the visual field as the animal is flying, or when it is hanging under the water surface. Within these zones, the rhabdomeres of the two central photoreceptors of each ommatidium are untwisted (and therefore polarization sensitive), yet their microvilli orientations differ between zones: the two most ventrally facing zones are formed by ommatidia containing photoreceptor pairs with orthogonally oriented microvilli, a structure perfectly adapted for detecting polarized reflections like water surfaces in a way that is insensitive to fluctuations in radiant intensity (Schwind, 1983b). Keeping in mind the optical axes of the photoreceptors in question, it appears therefore that *Notonecta* uses orthogonal analyzers to detect water surfaces when flying. This design is therefore similar to the fan-shaped array of orthogonal analyzers in the DRA. Orthogonally oriented rhabdomeric microvilli were also proposed for the ventralmost ommatidia of the non-biting midge *Chironomus transvaalensis*, yet to our knowledge no 3D reconstruction was performed to demonstrate an increased polarization sensitivity (Lerner et al., 2008). The third, most dorsally located zone of ventral *Notonecta* ommatidia right adjacent to the “main” retina, contains photoreceptor pairs with more or less parallel rhabdomeric microvilli—a design that may increase contrast during under water vision, while the animal is hanging under the water surface (a theory supported by the optical axes of these photoreceptors; Wehner, 1987, 2001). Overall, such an interrupted design in which the ventral periphery of the retina is subdivided into discrete zones represents an ideal adaptation to the sum of its very specialized aquatic lifestyles above and below the water surface, all of which are directly affected by horizontally polarized light.

A completely different retinal design was described for long-legged flies (Dolichopodidae), which live close to water bodies, and are known to hunt smaller insects on the water surface (Figures 3J–N). The retina of several Dolichopodidae species consists of alternating rows of ommatidia that can be identified based on their orange/red (Type A) vs. green/yellow (Tybe B) reflecting lenses (Stavenga et al., 2017). More importantly, the rhabdomeric ultrastructure of two central photoreceptors (called R7 and R8 in related *Drosophila*) differs between alternating rows of Type A and Type B ommatidia: the rhabdomeric microvilli of “Type A” central photoreceptors are both aligned along the dorsoventral axis, whereas an orthogonal orientation is found in Type B ommatidia (Trujillo-Cenóz and Bernard, 1972). It appears therefore, that “Type A” ommatidia would be perfectly suited for detecting objects against the water surface, by filtering out the horizontally polarized glare, whereas “Type B” ommatidia could be used to detect the water bodies themselves. Additionally, the different modes of polarization sensitivity could be used for perceiving “false colors”, since the two inner photoreceptors might express different Rhodopsin molecules. It remains unknown how signals from intermixed, yet alternating rows of ommatidia with different functional properties could be processed by post-synaptic units. Nevertheless, the problem is similar to the integration of signals from stochastically distributed ommatidial subtypes in other dipteran species (like *Drosophila* or* Musca*), as we will discuss in the “Neural Circuits Connecting to Specific Ommatidial Subtypes—Lessons from *Drosophila*” section. Taken together, retinal specializations in the ventral retina that are most likely related to polarized reflections (based on rhabdomeric ultrastructure) can serve very different functions, depending on their arrangement, their optical axes and the lifestyle of the insect: attraction to water via detection of horizontal e-vectors, the specific screening of such surface-reflected light, or even underwater vision. In some cases several of these functions appear to be integrated within one and the same retina.

In addition to these three specific examples for polarization-sensitive photoreceptors being organized in specific regions outside the DRA, several examples exist where insect photoreceptor subtypes throughout the retina seem to partially or completely untwist. Recently, photoreceptor cells with extreme polarization-sensitivity were characterized in the European corn borer moth *Ostrinia nubialis* (Belušič et al., 2017). In this case, each ommatidium contains one or two blue-sensitive photoreceptors with straight rhabdomeric microvilli manifesting polarization-sensitivities far greater than those measured in the DRA of the same animal. Interestingly, a very similar retinal design seems to have evolved independently in some scarab beetles (Gokan, 1989). Although the functional role of these extremely polarization-sensitive cells is not yet understood, the orientation of their rhabdomeric microvilli along the dorsoventral axis has led to the hypothesis that they could be used for filtering out horizontally polarized reflections, or for detecting vertically polarized skylight patterns in the north and south at sunset or sunrise. Less dramatic examples where photoreceptor subtypes manifest only partial untwisting of their rhabdomeric microvilli exist for several species. Such a design must result in mixing of e-vector information with the perception of either color or intensity. For instance, the “false color” detection system of the Australian orchard butterfly *Papilio aegeus* results from blue- and green-sensitive photoreceptors outside the DRA retaining polarization sensitivity due to insufficient rhabdomere twist (Arikawa and Uchiyama, 1996). Hence, the polarized reflections from different leaves (and therefore potential oviposition sites) will be perceived as different colors as the animal flies by. Another example is from blood-sucking horse flies (Tabanidae): electron microscopy revealed that in the mid region of the retina, both R7 and R8 cell rhabdomeres are largely untwisted, a design that should also function as a “false color” system. It is therefore possible that tabanid inner photoreceptors could be used for finding prey via the polarized light reflected off their fur (Wunderer and Smola, 1986; Smith and Butler, 1991). Interestingly, very similar studies also identified a subtype of untwisted R8 photoreceptor in blow flies, yet no specific function could be attributed to it (Wunderer and Smola, 1982). Similarly, systematic analysis of rhabdomere twist in *Drosophila melanogaster* revealed a low number of untwisted, UV-sensitive R7 cells in the ventral fly retina (Wernet et al., 2012). Together with low twisting R1–6 photoreceptors within the same ommatidia, these cells could provide the retinal substrate for *Drosophila*’s polarotactic responses to linearly polarized stimuli presented (Wolf et al., 1980; Wernet et al., 2012; Velez et al., 2014a,b). The exact number and distribution of untwisted R7 cells remains unknown and additional studies are needed for a complete description of a putative “ventral polarization area” formed by these cells somewhere in the fly retina. It must be noted that the analysis of rhabdomere twist is tedious and labor intensive, due to the need for 3D reconstruction of serial electron microscopy sections. It is therefore possible that polarization-sensitive photoreceptors might exist in the ventral periphery of the retina of many insect species, yet it is likely that they have been overlooked in the past.

## Neural Circuits Connecting to Specific Ommatidial Subtypes—Lessons From *Drosophila*

**Figure 4:**
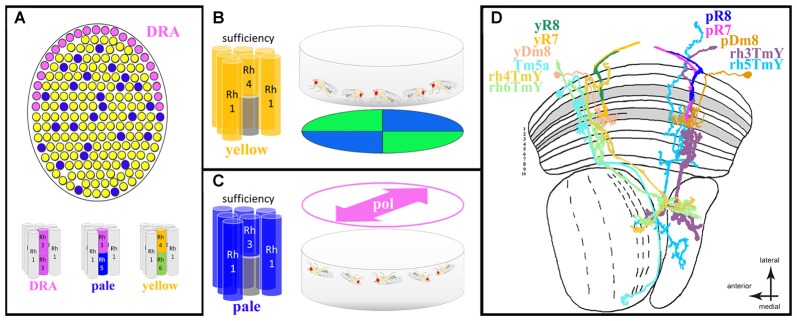
*Drosophila* ventral polarization vision and ommatidial subtype-specific circuitry. **(A)** Schematic description of the *Drosophila* retinal mosaic: specialized, polarization-sensitive ommatidia for detecting celestial cues are found in the dorsal rim area (DRA; pink), where both inner photoreceptors (R7 and R8) express the UV Rhodopsin Rh3. The remaining retina is populated by randomly distributed omatidial subtypes named “pale” and “yellow” in which R7 and R8 cells always express specific Rhodopsins. All short visual fiber photoreceptors R1–6 express the same Rhodopsin (Rh1) across all ommatidial subtypes. **(B)** Summary of population responses of walking *Drosophila* trained to discriminate between colors (blue and green quadrants) observed with the ventral half of the retina. Sufficiency experiments using cell-type specific restoration of the phototransduction cascade component NorpA (Phospholipase D) revealed a specific role for “yellow” ommatidia (more specifically: the combination of Rh4-containing yR7 cells and R1–6 photoreceptors). **(C)** Summary of spontaneous alignment behavior of (upside-down) walking *Drosophila* populations in response to linearly polarized UV light perceived with the ventral half of the retina. In this case, sufficiency experiments using cell-type specific NorpA rescue revealed a specific role for “pale” ommatidia (more specifically: the combination of Rh3-containing pR7 cells and R1–6 photoreceptors). **(D)** Summary of our current knowledge about ommatidial subtype-specific neural circuit elements within the optic lobes of *Drosophila*. In the distal medulla neuropile, both pR7 and yR7 cells connect to the amacrine-like cell type Dm8. Results obtained from RNA profiling of inner photoreceptors and Dm8 cells suggests expression of specific cell-surface molecules in yR7 cells (expressing Dpr11) and their Dm8 connections (termed here yDm8, expressing DIP-γ) are important for establishing pale vs. yellow specific circuits. Furthermore, anatomical studies have identified the transmedullary (Tm) cell type Tm5a (sending projections to the lobula neuropile) as being specific to columns connected to yellow ommatidia (hence missing from those columns connected to pale ommatidia). Finally, a study using a trans-synaptic tracer technique identified four distinct classes of bifurcated TmY cell types (sending projections to both the lobula and lobula plate neuropiles), each type being specifically connected to pR7, yR7, pR8, or yR8 cells, respectively (hence termed rh3Tmy, rh4Tmy, rh5TmY and rh6TmY).

The ommatidial mosaic of the fruit fly *Drosophila melanogaster* has long served as a powerful genetic model system for the dissection of cell-cell interactions during photoreceptor cell fate specification, revealing a long list of molecular key players involved in this process (Johnston, 2013; Wernet et al., 2014). Of particular interest are transcription factors expressed in very restricted groups of cells where they induce specific cell types while repressing other fates. For instance, the homeodomain transcription factor Homothorax (Hth) is expressed specifically in developing polarization-sensitive central photoreceptors R7 and R8 exclusively within prospective DRA ommatidia which form a narrow band of ommatidia along the dorsal margin of the fly eye (Wernet et al., 2003; Wernet and Desplan, 2014). Genetic manipulations revealed that Hth is both necessary and sufficient to induce the DRA fate when (mis-) expressed in any inner photoreceptor (Wernet et al., 2003). Importantly, Hth is usually not expressed at the ventral margin of the retina, nor anywhere else in the retina where one could suspect polarization-sensitive photoreceptors. The rest of the fly retina consists of two randomly yet unevenly distributed ommatidial types called “pale” and “yellow” (summarized in Figure 4A). The main difference between these two subtypes lies in the identity of the Rhodopsin molecules expressed by the central photoreceptors R7 and R8, resulting in subtype-specific pairing of the Rh3/Rh5 gene products in “pale” ommatidia and Rh4/Rh6 in “yellow” ommatidia (where both Rh3 and Rh4 are UV-sensitive Rhodopsins, Rh5 is blue-sensitive, and Rh6 is UV+green-sensitive; Johnston, 2013). Due to this mosaic of randomly distributed chromatic sensitivities it was long assumed that pale and yellow ommatidia serve color vision in *Drosophila*, and several recent studies have supported this hypothesis (Yamaguchi et al., 2010; Schnaitmann et al., 2013, 2018; Melnattur et al., 2014). Importantly, very similar ommatidial mosaics with two or three randomly distributed ommatidial subtypes have been described for many different insect species (Diptera, Hymenoptera, Lepidoptera, Hemiptera, Orthoptera; reviewed in: Wernet et al., 2015). More importantly it was recently shown that the same transcription factor is responsible for establishing the pale/yellow mosaic between central photoreceptor cells, both in flies and butterflies: using the Crispr/Cas9 technique in *Papilio* butterflies to produce large patches of retina lacking the Dioxin receptor (called Spineless in *Drosophila*), the butterfly retinal mosaic was disrupted in a predictable manner (Perry et al., 2016). Like in the *Drosophila* retina, loss of Spineless led to a complete loss of one ommatidial subtype (“yellow” in *Drosophila*, Wernet et al., 2006). It appears therefore, that the molecular mechanisms shaping the stochastic retinal mosaic are conserved between these distantly related species.

A rather unexpected potential function of the randomly distributed “pale” and “yellow” ommatidia as separate input channels for polarization vision vs. color vision was revealed by two independent studies both presenting visual stimuli to isogenic populations of walking *Drosophila*. In both cases sufficiency of “pale” and “yellow” ommatidia was investigated by rescuing phototransduction in a cell-type specific manner in blind flies lacking an eye-specific isoform of Phospholipase C (called NorpA in *Drosophila*). One study found that “yellow” ommatidia were sufficient to mediate color discrimination in an assay where the flies were presented blue and green quadrants (more specifically: the combination of *rh4*-expressing yR7 cells in combination with *rh6*-expressing yR8 or in combination with R1–6, the short visual fiber photoreceptors; Schnaitmann et al., 2013). In this assay, “pale” ommatidia were not sufficient to mediate color discrimination (Figure 4B). Another study presented isogenic populations of walking *Drosophila* with linearly polarized light of different, fixed e-vector orientations (Wernet et al., 2012). Strikingly, in this case only rescue of “pale” ommatidia was sufficient to mediate a polarotactic orientation response in which the flies oriented their body with the incident e-vector (more specifically: the combination of *rh3*-expressing pR7 cells with R1–6 photoreceptors). However, “yellow” ommatidia were not sufficient to mediate such a response (Figure 4C). These genetic experiments indicate that under certain conditions “pale” and “yellow” ommatidia in the ventral half of the retina may serve two separate functions: color vision (yellow) vs. polarization vision (pale). If this was the case, one would predict differences in rhabdomeric twist between pR7 (not twisting) and yR7 (twisting) cells. However, the analysis of rhabdomeric twist within a randomly chosen region of the fly retina revealed no difference between the two (both twisting) and the low-twisting R7 cells that were identified could not be attributed to a specific subtype (Wernet et al., 2012). A possible functional specialization therefore cannot apply to all “pale” vs. “yellow” ommatidia. However, there may exist a region within the ventral half of the fly retina where pR7 cells are specifically untwisted while yR7 cells remain twisted. An example for such subtype-specific untwisting of photoreceptor rhabdomeres was described for R8 cells in *Calliphora*, a functional significance for this anatomical substrate has yet to be demonstrated (Wunderer and Smola, 1982). Taken together, groups of ommatidia from different subtypes may form segregated input channels mediating distinct behavioral responses (color vs. polarization vision), yet more data is needed to understand their relative contribution. Strikingly, functionally specialized ommatidial subtypes could be distributed randomly (as in the case of *Drosophila*), or in alternating rows (as shown for *Dolichopodidae*)—two fundamentally different design principles that could be viewed as alternative solutions for spatially separating these inputs without sacrificing too much of the visual field to either one modality (while neglecting the other). Interestingly, similar segregation of color- and polarization sensitive pathways has recently been proposed for a vertebrate retina (Novales Flamarique, 2017).

Over the past few decades, the neural circuits mediating polarization vision downstream of DRA ommatidia have been described in great detail, across species. The circuit diagram deduced from these studies reveals how celestial e-vectors are represented in the central brain, how they are integrated with other positional cues like the sun, and how the compass system is compensating for the changes in e-vector orientation as the sun moves across the celestial hemisphere (Homberg et al., 2011; Homberg, 2015). In contrast, next to nothing is known about the neural circuits underlying ommatidial specializations in the ventral periphery of the insect retina, like those described for *Gerris*, *Notonecta* and *Dolichopodidae*. In recent years, powerful molecular genetic tools have been developed for the cellular dissection of neural circuits across the *Drosophila* visual system, with a special emphasis on the optic lobes (Takemura et al., 2015). One first step towards addressing the neural circuitry of non-celestial polarization vision therefore lies in identifying optic lobe cell types that make ommatidial subtype-specific connections. At first glance, it seems hard to imagine how such connections could be wired during development of the visual system, given that *Drosophila* “pale” and “yellow” ommatidia are specified in a stochastic and therefore non-deterministic manner. Nevertheless, examples for pale- vs. yellow-specific optic lobe cell types exist and are currently increasing. For instance, anatomical characterization of the transmedullary cell type Tm5 (connecting the medulla neuropile with the lobula neuropile) revealed three subtypes termed Tm5a, Tm5b and Tm5c (Meinertzhagen et al., 2009). Interestingly, Tm5a cells were found to specifically arborize dendrites in single medulla columns containing yR7 terminals (Karuppudurai et al., 2014), whereas Tm5b and Tm5c are not subtype-specific (summarized in Figure 4D). Another study revealed subtype-specific circuit elements using a transgenic approach for trans-synaptically labeling optic lobe cell types that are connected to specific photoreceptor subtypes (Jagadish et al., 2014). In this case, four similar yet different cell types of so-called TmY cells with bifurcated axons (connecting the medulla neuropile with both the lobula and lobula plate neuropiles) were identified. Each of the four TmY subtypes appeared to specifically connect to either pR7, yR7, pR8, or yR8 cells and they were therefore termed rh3-TmY, rh4-TmY, rh5-TmY and rh6-TmY. So far, the existence of these cells and their subtype specific synaptic connections remain to be confirmed by EM reconstruction (Takemura et al., 2015). If confirmed, it is not known how these cell types would establish specific connections with photoreceptor cells that were specified stochastically. However, an important first step towards understanding how such wiring could be achieved came from two studies investigating the development of R7 connections with their most important synaptic partners, a distal medulla cell type called Dm8. Roughly every medulla column contains one Dm8 cell that receives inputs from ~10–16 neighboring R7 cells (Gao et al., 2008; Karuppudurai et al., 2014; Ting et al., 2014). Assuming that each Dm8 cell receives preferential synaptic input from the R7 terminal located within its “home cartridge”, one can therefore deduce the existence of Dm8 cells that receive predominant “pale” vs. “yellow” input (hence termed pDm8 and yDm8, in Figure 4D). How “pale” and “yellow” information is then processed further is currently not well understood and made more difficult by the fact that Dm8 cells appear to be locally processing units without a clear directed axonal output (Gao et al., 2008; Karuppudurai et al., 2014; Ting et al., 2014; Lin et al., 2016). Via profiling of the RNA transcriptome of R7 vs. R8 cells, recent studies studying the role of two classes of immunoglobulin transmembrane proteins (called DIPs and Dpr’s) identified one protein that is specifically expressed in developing yR7 cells (Dpr11). More importantly, its ligand DIP-γ, the protein that specifically binds to Dpr11, is expressed in developing Dm8 cells (Carrillo et al., 2015; Tan et al., 2015). It appears therefore that the specific interaction between these transmembrane proteins could be the key to establishing subtype-specific connectivity between stochastically specified photoreceptor subtypes and their specific post-synaptic targets, thereby shaping distinct input pathways with different properties. Although still being in a very early stage, these experiments on ommatidial subtype-specific wiring could serve as a model system for understanding how neural circuits in the ventral periphery of the insect retina are shaped in order to result in functionally specialized channels.

## Concluding Remarks

Different visual responses of insects to linearly polarized reflections have been described. Given the general importance of water bodies as habitats for insects, as well as the well-described adaptation of many species to a (semi-)aquatic lifestyle, a multitude of such behaviors could have been expected. That makes it even more surprising that only few examples exist for the retinal detectors responsible for processing linearly polarized reflections. The most fascinating aspect of these retinal detectors remains their developmental Bauplan: specialized ommatidia are found either restricted locally at the ventral edge, or subdivided into zones or even alternating stripes. Some retinal designs are capable of detecting linearly polarized reflections (the zonated ventral retina of Notonecta, or those ommatidial rows of Dolichopodidae with crossed polarizers), whereas others most likely serve to filter out linearly polarized light, like glare at the water surface (for instance the ventral retina of the water strider and potentially the retina of the corn borer moth). In the future, new studies should focus on analyzing the retinal ultrastructure from additional (semi-)aquatic insect species to deepen our understanding of how linearly polarized reflections are being detected.

What are the neural circuits processing the information from these ommatidial subtypes? To our knowledge, nothing is known about the underlying circuits in (semi-)aquatic insects. Using electrophysiology, many of the underlying circuit elements can be characterized. We expect that future studies on different species like tabanids will reveal important insight into the functional properties and the anatomy of the underlying circuits. Alternatively, we have shown how the investigation of photoreceptor subtype-specification in the molecular genetic model organism *Drosophila melanogaster* can provide insight into how the establishment of ommatidial subtype-specific circuitry may be regulated. A growing number of studies demonstrates the existence of neural circuit elements whose identity or morphology are specific to either one of the two stochastically distributed ommatidial subtypes. Combining the molecular genetic toolkit of *Drosophila* with behavioral paradigms for quantifying the behavioral response to linearly polarized reflections therefore presents another attractive approach for studying how subtype-specific cell types might specifically alter the function of repetitive, retinotopic micro-circuits.

## Author Contributions

MFW wrote the manuscript. TH and JU assisted with the writing and provided all the figures.

## Conflict of Interest Statement

The authors declare that the research was conducted in the absence of any commercial or financial relationships that could be construed as a potential conflict of interest.
